# The Treatment of Surgical Tuberculosis by Vaccines1Read at the annual meeting of the Medical Society of the State of New York at Albany, N. Y., January 26, 1910.

**Published:** 1910-08

**Authors:** James A. Macleod, Norman K. Macleod

**Affiliations:** Buffalo, N. Y. 327 Delaware Avenue; Buffalo, N Y. 327 Delaware Avenue


### The Treatment of Surgical Tuberculosis by Vaccines[1]

BY JAMES A. MAC LEOD, M.D., M.R.C.S.
Buffalo, N. Y.
and
NORMAN K. MAC LEOD, M.D.
Buffalo, N Y.

IN the scope of this paper it is an almost insurmountable difficulty to attempt to correlate and condense the facts that are necessary for a fit discussion of the subject which we propose to place before this meeting. Since Koch first announced his great discovery, investigators from all quarters have poured into literature the fruits of their investigations. This vast amount of

1. Read at the annual meeting of the Medical Society of the State of New York at Albany, N. Y., January 26, 1910.

work, resulting in no definite goal, has had its origin in the fact that the tubercle bacillus has not conformed to the lines followed by the great mass of bacteria. Such being the case let it be granted: first, that up to the present we have no exact scientific knowledge of the true toxins elaborated by the bacillus. *Second,* that we have no conception whatever of the immunity response offered by the body to combat an invasion by the organism. *Third,* that we are not even certain whether the body enjoys a general or but a local resistance toward the tubercle bacillus.

Tuberculosis is essentially a local disease; but is the resistance offered to it by the body a general one, that is one lying in the blood itself, or a local one, that is, one lying in the tissues themselves, or a combination of these two? The point at issue is of far greater importance than would at first appear, since it has a direct bearing upon the great problem that confronts us. The probability is that the combination of the two holds true, otherwise contradictions exist. Thus tubercle bacilli themselves, and by this we refer to the human and bovine types particularly, are in our present knowledge among the most highly organised in the bacterial world. It would seem, according to some authorities, that these two types are the result of an evolution, which has raised them from the ranks of the lowest non-pathogenic forms of acid resistant organisms, such as are found everywhere in nature, through hosts of increasing complexity until the present parasitic state has been attained. It is due to this high degree of organisation that, after years of study, the pathologists are forced to the conclusion that specific therapy is yet far off. Infections with the tubercle bacillus are local ones, as we have pointed out above, and except in certain conditions they do not, per se, produce temperature. Yet from the local foci toxins must be absorbed, otherwise no deleterious effects could ensue. What the toxin is we do not know, nor has it ever been obtained; Marmorek's serum obtained from the growth of the bacilli in a leucotoxic medium is said to contain this toxin, but this has not been proved. Tuberculin, it is said, does not contain it, and yet it has a known curative value. Since Koch's first announcement of his discovery of the tubercle bacillus every conceivable method has been exploited in an endeavor to produce either an active or passive state of immunity or resistance towards the organism. Von Behring, following Koch's announcement that there were two distinct types of bacillus, the human and bovine, declared that in his bovovaccine he had discovered Jennerisation as applied in his experiments to the raising of an immune state in calves towards the bovine forms of tuberculosis. Unhappily recent announcements show clearly that no immune state exists, only a resistance lasting over a short variable space of time, to be followed by a state of increased susceptibility. That no immune state exists is proved by the recovery of living virulent organisms from the lymph glands of the animal inoculated. Jennerisation has failed, pasteurisation has failed, the various sera have failed. Tuberculin alone has given a certain measure of success. What the action or reaction of the various tuberculins is we do not know for certain, but it is believed that the action is upon the tuberculous tissues and not directly upon the bacilli themselves. That there is an anti-tuberculin present is undoubted since Wassermann has demonstrated that complement fixation takes place in tubercular infections, but it has not been proved that this substance is bactericidal. Nor have the agglutins, upon which some diagnostic value has been placed, so far been shown to inhibit the disease. It is so with all other bodies with the one possible exception—the opsonins. Wright and Douglass claimed that these opsonins were bactericidal, and showed that by increasing their content in the serum the phagocytic power of that serum was raised. They deduced from this that opsonins were directly responsible for immunity to certain diseases, although such a role cannot be placed entirely to the credit of these bodies, yet certainly the fact remains that subsequent to inoculation with tuberculin the opsonic content of the blood is increased. With this increase clinical improvement is in many cases manifested. Whether this improvement is due to the opsonins or simply to a local hyperemia cannot be proved. Bullock makes the statement that cases of lupus with a high opsonic index give better results with *x*-ray treatment than those cases which possess a low index. He further states that these later cases, upon their index being raised, also do more satisfactorily. This does not prove that opsonins are responsible for this improvement, but that their estimation gives some indication of the resistance, whatever it may be, that is offered by the body against the infection, If we consider then the opsonins as a measure, not of the immunity but of the general resistance offered, we have a practical foundation to work upon. This foundation is possibly an empirical one but it is the only one presenting itself to us. Agglutination tests have been and are employed, but since these bodies are frequently absent in the disease, their use, as governing tests, seems inadequate. Having defined this empirical position, upon which our ideas rest, we may be permitted to approach closer to a practical consideration of the use of tuberculin as applied to surgical tuberculosis. In the combination of treatments, surgical and laboratory, we have kept constantly before us this viewpoint—the improvement of our patient. In our attempts to prove our contentions we have employed only comparative deductions; that is, a comparison between our former surgical results without the use of tuberculin, and of our present surgical results with the use of tuberculin.

We will not enter into the question of the general pathology of the surgical forms of tuberculosis in this discussion, and will but give passing notice to the bacteriology of it. It is becoming the consensus of opinion among the bacteriologists of today that the tuberculosis found in the human body and in different animals is caused by the same organism, variously altered in character by its prolonged habitat in its various hosts. It may be taken as well established that the disease occurring in the human body is caused by either two of these approximate types of the organism. First, the human type of the tubercle bacillus; second, the bovine type of the tubercle bacillus. These two types can be definitely differentiated by appropriate laboratory methods, with which we will not concern ourselves in this discussion. The human type of the bacillus is the one more commonly found in the adult, that is, in the tuberculosis of the lungs. The bovine type of the bacillus is the one more commonly found in children, that is, in the surgical forms of the disease. The two types may, however, be co-existent in the same patient. It is interesting to note that the two types may be found in the same location under different circumstances—namely, in laryngeal tuberculosis, we find that when the disease is primary it commences in the deeper layers of the mucous membrane, and is usually bovine in its type; when the disease is secondary to tuberculosis of the lungs it commences as a superficial ulceration or erosion of the mucous membrane, and the type is similar to that causing the pulmonary tuberculosis, which is more commonly human in its type.

We will not enter into the question of the diagnosis of the surgical forms of tuberculosis in this discussion, except to say that we have used in our practice the various tests for the detection of early or suspected tuberculosis, and have found them to be fairly reliable, when taken in conjunction with the clinical symptoms and the physical signs.

Without further preface we will direct your attention to the problem of treatment, and we will deal at some length with the question of the raising of the resistance of the patient with tuberculin. It is not necessary for us in addressing this body to deal with the surgical methods and measures to be employed, but at the same time we wish to impress upon you that the raising of the resistance of the patient is but an aid to sound surgical procedure. The problem of treatment is a large one and can best be approached by dividing it into classes and discussing each class by itself.

1. The kind of tuberculin to be employed.
2. The estimation of the dose of the tuberculin.
3. The surgical remedies.
4. The question and time of the operation.
5. The treatment following the operation.
6. The treatment of cases which have been operated upon without preliminary treatment with tuberculin, and followed by the formation of sinus or sinuses.

1. *The kind of tuberculin to be employed.*—In our practice we use only the emulsion of the dead tubercle bacilli and in speaking of tuberculin or vaccine in this paper we wish it to be understood that this preparation is meant. From our experience in the treatment of other bacterial infections one would think that a specific or autogenous vaccine would be the one to employ in the treatment of these patients. It is usually difficult, and in many cases impossible, to isolate the specific infecting organism in the surgical forms of tuberculosis; moreover, it has been found by experience that an appropriate stock vaccine fulfils the requirements of the case, just as in the case of staphylococcus and other infections. Experience with stock vaccines, however, demonstrated that the patient was most benefited by a vaccine prepared, not frcm the bacillus of the type causing the disease, but from the bacillus of the other type. This is in direct variance with our experience in the treatment of other bacterial infections, and we are not prepared at present to offer an explanation. The differential diagnosis of the two types is possible by appropriate methods, but as it requires a great amount of time and work it is seldom done, except for the sake of special scientific interest.

Raw, of Liverpool, made the statement both in this country and abroad that all cases of localised tuberculosis were due to the bovine type of the bacillus, and that only pulmonary tuberculosis was due to the human type of the bacillus. He further stated that the two forms could not exist in the body at the same time, because the infection with the one immunised the body against the other. These statements were seized by workers in tuberculin with delight, since it made the diagnosis as regards the type of the infection extremely simple. Unfortunately other investigators have flatly denied these statements of Raw, and have proven beyond question that although localised tuberculosis in a large percentage of cases is due to the bovine type of the bacillus, yet the human variety is responsible for a considerable number of cases. Early in our experience we accepted Raw's teachings and used a vaccine prepared from the human type of the bacillus in our treatment of the surgical forms of the disease. In those cases, however, which did not appear to be progressing as well as we expected, we changed to the bovine tuberculin with benefit, so we thought, to our patients. This improvement, and in many cases it was marked, pointed strongly to an erroneous diagnosis as to the type having been made. It was, however, noted under the administration of the first vaccine that although the local focus of the disease was not perceptibly altered, the general condition of the patient was, if anything, slightly improved.

The difficulty in making an absolutely correct diagnosis as we use only the emulsion of the dead tubercle bacilli, and in that the use of the vaccine, of the same type as that causing the actual focus, does not seem to have any harmful effect on the patient, led us some twelve months ago to the employment of a mixture of the two vaccines, as advocated by Allen, in all of our cases, irrespective of the type of the bacillus causing the disease. Our results have been most gratifying. If on continued further trial in the use of the mixed vaccine our results warrant us in their continuance it will be of the greatest service in our treatment of these cases. It will make the question of diagnosis much simpler, as the need of accurate differential diagnosis between the two types of the bacillus will be abolished, except for the sake of special scientific interest.

2. *The estimation of the dose of the tuberculin.*—When tuberculin was first introduced by Koch and used by the profession there was no method by which the dose could be estimated. Indiscriminate dosage was the result, and in nearly all cases large doses were the rule. The reactions following the use of the tuberculin were marked; the results were unsatisfactory and in many cases disastrous, in fact so much so that the therapy soon fell into disrepute and was practically discarded by the surgical profession. In the use of tuberculin the dose is the all important factor. This holds true not only when the patient is being inoculated with tuberculin, but also when auto-inoculation is being practised; regarding the latter we refer to passive movements. In considering the dose of tuberculin to be employed the following points must be studied.

A. The personal equation.
B. The site of the lesion.
C. The state of the health of the patient.

D. The age of the patient.
E. The question of autoinoculation.
F. The opsonic index to the tubercle bacillus.

A. *The personal equation.*—Occasionally upon inoculation a violent reaction follows upon a dose which, under ordinary circumstances, would be attended by no untoward symptoms. This is possibly due to a personal idiosyncrasy, or more probably, if we are to believe the theory of Calmette as to the production of reaction following inoculation, to the setting free of toxins from a lesion that is, if we may be allowed the license, in an unstable state. The patient in other words is in a state of increased susceptibility; this susceptibility is a local one confined to the lesion itself, the general signs being due to toxins liberated as the result of the reaction. From this point one may deduce the truth, that the earlier the lesion the smaller the initial dose. Old standing cases tolerate larger doses than do the more recent ones.

B. *The site of the lesion.*—This especially applies to tuberculosis of the epididymis and kidney, as patients suffering from an infection of these organs do not tolerate as large doses as do patients suffering from infections of other parts of the body. Formerly we made it a practice not to inoculate in tuberculosis of the kidney unless the other kidney was unaffected. The dangers of causing a marked reaction in a diseased secreting organ is apparent. At the present time, if there seems to be any hope of overcoming the disease, we inoculate, but only with extremely small initial doses, increasing with caution. We feel although a certain amount of hyperemia accompanies even the smallest of doses, that it is of so mild a character that no harm can be done.

C. *The state of the health of the patient.*—Tuberculin is one of the most powerful drugs at our command. The stimulation following its use is sharp and strong. Its first effect is to paralyze the cells; this passes off and is followed by a true stimulation, whereby the cell production is increased. In order to respond to this stimulation the body cells must be in as fit a condition as it is possible to place them; with this in mind every possible attention should be paid to the hygienic surroundings and general condition of the patient. It was demonstrated during experiments on animals that vaccinated calves during the vaccinal period were more prone to tubercular infection than non-vaccinated ones. Bearing this in mind it is wise to specially guard our patients during treatment from sources of fresh infection.

D. *The age of the patient.*—Just as in the prescribing of other strong and powerful drugs the age of the patient is an important desideratum in our estimation of the dose of tuberculin. Children tolerate a much larger dose, comparatively speaking, than do adults.

E. *The question of autoinoculation.*—Of all considerations of the estimation of the dose this is the most important. It is obviously impossible to estimate the dose of the tuberculin to be administered, if the patient is constantly by active movement giving himself irregular doses at irregular intervals of what practically amounts to the same thing. The old surgical dictum, "That an infected part must be put at rest" is more true in the employment of the vaccine than in any other therapy. Autoinoculation must be reduced to a minimum by appropriate surgical methods if we are to hope for successful results. If the clotting power of the blood be low, as demonstrated by the increased clotting time, calcium lactate should be administered to lessen absorption from the focus of the infection.

F. *The opsonic index to the tubercle bacillus.*—In our work we employ both the opsonic and fractional dose methods. Many claim that the opsonic index is of no value. It is quite true that it has not the great practical value that was at first claimed for it. It is quite true that it is subject to a considerable error, but very little more, if any, than other clinical estimations. The index has played a most important part in the past, and still enjoys a considerable value. In difficult cases, in mixed infections as with the bacillus coli communis, in diseases of the genito-urinary tract, in those cases in which it is desirable to obtain a sufficient response with the smallest possible dose, the index is of considerable value. In those cases which possess no particular difficulties, the fractional method is indicated; it requires close attention and experience if results are to be obtained.

In treatment with tuberculin "haste" must be avoided; results are slow. The resistance should be raised and tolerance established to the drug gradually. After each inoculation a reaction follows in the local lesion, but this should be of so mild a character as to be, in the earlier doses, not noticeable. Formerly it was the practice to administer large doses at long intervals, but we believe that more benefit is to be gained by small doses at short intervals. In our cases we have seldom found it necessary to give a larger dose than one to four thousandth part of a milligram of the combined tuberculins. The interval between inoculations ranges between five and seven days.

3. *The surgical remedies.*—We will not discuss in this paper, as stated above, the question of the surgical methods and measures to be employed in the treatment of the surgical forms of tuberculosis: let us take it as granted that such are given their proper and most important position in the total question of the treatment of the condition.

4. *The question and time of operation.*—In those cases where operation is necessary the institution of inoculations prior to the operation is of the greatest value. The benefit to be gained by this is too great not to be emphasised. In tubercular glands of the neck especially does this apply. It is impossible in this condition to remove all of the glands. The surgeon aims at removing those in sight, and relies on the body to overcome the disease in the small remaining ones. If this obtains a beautiful result is procured. But how often do we see several unsightly scars upon a neck, and how often do we see sinuses persist after operation? We believe that with efficient prior inoculation these dangers are eliminated. In regard to treatment, glands of the neck may be divided into three classes:

*First.*—Those cases in which recovery is possible without operation.

*Second.*—Those cases in which the necessity of operation is probable.

*Third.*—Those cases in which caseation has occurred and progressed to liquefaction and in which operation is inevitable.

*First.*—In this class of cases the glands are small, discrete and not perceptibly matted together. There is no evidence of breaking down. This class does remarkably well under tuberculin treatment.

*Second.*—In this class of cases the glands are large and very much matted together. The process is usually too extensive to be cured by tuberculin unaided by operation. The line of procedure to be followed is to inoculate over a considerable space of time. The glands must be kept under constant observation to instantly forestall by operation any extensive softening. This class, rarely, if ever, arrives at anything but a most satisfactory termination.

*Third.*—In this class of cases operation is inevitable, and the procedure that we follow is to aspirate when possible. If we can by this means and by the use of tuberculin control the breaking down until we have the glands in a favorable condition for operation, we do so; but if the process is progressive, in spite of these means, early operation is imperative. In tuberculosis of the epididymis the same procedure may be applied. Prior to any operative interference it is wise to estimate the clotting power of the blood, and if low, administer calcium lactate in order to lessen as much as possible the autoinoculation following the traumatism of the operation. Bearing in mind that the first effect of the tuberculin, as pointed out above, is paralysis of the cells, it is wise to wait until that effect has given place to the true stimulating effect before performing any operation, that is, wait until the patient is at or about the climax of the positive phase.

5. *The treatment following operation.*—When the autoinoculation caused by the traumatism of the operation has become reduced to a minimum, inoculations with the vaccines should be reinstituted and continued for at least six months. We find that in patients treated with the tuberculin prior to operation the wounds heal promptly with very little tendency to a secondary infection. Where, however, secondary infection does occur it quickly responds to inoculations with the appropriate vaccines.

6. *The treatment of cases which have been operated upon without preliminary treatment with tuberculin, and followed by the formation of a sinus or sinuses.*—In this class of cases the sinus or sinuses are lined with a thick pyogenic membrane, and we have, in addition to the tubercular infection, secondary infecting bacteria. Smears and cultures should always be taken to determine the secondary infection, and appropriate vaccines prepared and administered. The clotting power should be estimated, and if high it should be lowered by the administration of citric acid in order that the serum may have better access to the focus of the disease. If, after several weeks of inoculations with the tuberculin and the appropriate bacterial vaccines, and after the use of iodine and agents to promote osmosis, there is not distinct improvement it is usually necessary to thoroughly curette away the thick pyogenic lining membrane, in order that the vaccines may have better access to the focus of the disease.

In conclusion we would say that, after a wide experience in the treatment of the surgical forms of tuberculosis, we are of the emphatic opinion that in tuberculin we have an invaluable aid. Soon after the inoculations are instituted the general constitutional condition of the patient is materially improved; he feels better, he appears better, and he soon shows that he is really better by an increase in his weight. We would like to impress upon you that very marked improvement in the local disease is not to be looked for until after several months of inoculations. Where operations are performed upon patients, previously treated with tuberculin, the wounds heal, as a rule, promptly; the danger of secondary infection is much reduced, and where it does occur it quickly responds to the inoculations with the suitable vaccines; the dangers of a general systematic miliary infection and the dangers of lardaceous disease are practically eliminated.

327 Delaware Avenue.

